# The Use of NanoTrap Particles as a Sample Enrichment Method to Enhance the Detection of Rift Valley Fever Virus

**DOI:** 10.1371/journal.pntd.0002296

**Published:** 2013-07-04

**Authors:** Nazly Shafagati, Aarthi Narayanan, Alan Baer, Katherine Fite, Chelsea Pinkham, Charles Bailey, Fatah Kashanchi, Benjamin Lepene, Kylene Kehn-Hall

**Affiliations:** 1 National Center for Biodefense and Infectious Diseases, George Mason University, Manassas, Virginia, United States of America; 2 Ceres Nanoscience, Manassas, Virginia, United States of America; University of Texas Medical Branch, United States of America

## Abstract

**Background:**

Rift Valley Fever Virus (RVFV) is a zoonotic virus that is not only an emerging pathogen but is also considered a biodefense pathogen due to the threat it may cause to public health and national security. The current state of diagnosis has led to misdiagnosis early on in infection. Here we describe the use of a novel sample preparation technology, NanoTrap particles, to enhance the detection of RVFV. Previous studies demonstrated that NanoTrap particles lead to both 100 percent capture of protein analytes as well as an improvement of more than 100-fold in sensitivity compared to existing methods. Here we extend these findings by demonstrating the capture and enrichment of viruses.

**Results:**

Screening of NanoTrap particles indicated that one particle, NT53, was the most efficient at RVFV capture as demonstrated by both qRT-PCR and plaque assays. Importantly, NT53 capture of RVFV resulted in greater than 100-fold enrichment from low viral titers when other diagnostics assays may produce false negatives. NT53 was also capable of capturing and enhancing RVFV detection from serum samples. RVFV that was inactivated through either detergent or heat treatment was still found bound to NT53, indicating the ability to use NanoTrap particles for viral capture prior to transport to a BSL-2 environment. Furthermore, both NP-40-lysed virus and purified RVFV RNA were bound by NT53. Importantly, NT53 protected viral RNA from RNase A degradation, which was not observed with other commercially available beads. Incubation of RVFV samples with NT53 also resulted in increased viral stability as demonstrated through preservation of infectivity at elevated temperatures. Finally, NanoTrap particles were capable of capturing VEEV and HIV, demonstrating the broad applicability of NanoTrap particles for viral diagnostics.

**Conclusion:**

This study demonstrates NanoTrap particles are capable of capturing, enriching, and protecting RVFV virions. Furthermore, the use of NanoTrap particles can be extended to a variety of viruses, including VEEV and HIV.

## Author Summary

There is a dire need for fast and efficient diagnosis of many viral diseases. Our research specifically looked at RVFV, a virus that can only be worked with in biosafety level 3 (BSL-3) laboratories, and its capture with NanoTrap particles. NanoTrap particles are hydrogel particles that contain internal affinity baits. They have previously been used in the capture of several analytes, but never in the capture of whole virus particles. We were not only able to capture and detect RVFV at very low titers from both media and serum, but we were also able to inactivate the virus, which allows for its safe transport to BSL-2 laboratories. While there are other commercially available beads that can also capture virus, NanoTrap particles are the only beads that can protect the viral RNA from enzymatic degradation. Furthermore, we demonstrated that whole virus detection with NanoTrap particles is not limited to only RVFV, but that NanoTrap particles can be used to detect other viruses such as Human Immunodeficiency Virus (HIV) and Venezuelan Equine Encephalitis Virus (VEEV).

## Introduction

Rift Valley fever virus (RVFV) belongs to the genus *Phlebovirus* and family *Bunyaviridae*. RVFV is composed of a tripartite single-stranded RNA genome with large (L), medium (M), and small (S) segments [1], [2], [3], [4]. RVFV particles have icosahedral symmetry and are 90–110 nm in diameter [4]. The envelope is made up of a lipid bilayer that is embedded with the Gn and Gc glycoproteins. These glycoproteins, which are the most exposed components of the virus during infection, play a crucial role in the entry of the virus into the host cell.

RVFV is a highly pathogenic arthropod-borne virus that is primarily transmitted by mosquitoes, particularly after heavy rainfall. Although it can infect a wide range of vertebrate hosts, RVFV primarily affects livestock and humans [2]. Animals are infected through mosquito bites and other arthropod vectors. Humans are typically affected when they come in close contact with infected bodily fluids or tissues, but transmission via mosquito bites, as well as aerosolization may also occur. However, humans are dead-end hosts [1], [5].

Since being first identified in 1930 in the Rift Valley of Kenya, outbreaks have led to high mortality rates as well as significant economic loss [3]. RVFV has remained endemic in sub-Saharan Africa, causing major outbreaks throughout the continent over the last century [6]. In 1976, 200,000 individuals were infected and 600 fatal cases were reported in Egypt [5]. Most likely due to international livestock trade, it has since crossed the Arabian Peninsula into Saudi Arabia and Yemen. Over 30 mosquito species, mostly *Aedes* and *Culex* are vectors for RVFV [5]. Of particular concern is that the *Aedes* species is widely distributed in the EU countries and many of those countries (Turkey, Greece, Italy, Spain, Portugal, and France) have high-risk vector habitat areas that may serve as emergent sites. Moreover, in the Unites States, this species has been found in 23 states [7]. Since RVFV is capable of utilizing a wide range of mosquito vectors, the virus has the potential to spread further into non-endemic areas [5], [8].

Mortality rates are dependent on species and age. In livestock, mortality rates are as high as 30%. Mortality rates can reach as high as 95% in newborns and the young, while abortion rates are as high as 100% [5]. Symptoms in humans are usually mild and include febrile illness resembling the flu, with a small percentage developing serious clinical manifestations such as retinal lesions, meningoencephalitis, hepatitis, severe hemorrhagic fever, coma and death. In recent years an increase in mortality amongst humans from 2% to 45% has been reported, suggesting evolving mechanisms of virulence and mutations [3].

Due to its transmission via aerosolization, high pathogenicity, and classification as a Group III (bioterrorism potential) Category A emerging infectious disease by the NIAID, work with RVFV requires BSL-3 containment. It is highly suggested that laboratory staff working with RVFV be vaccinated. Therefore, diagnosis of RVFV is restricted to a small number of laboratories. This limitation has led to some delay in diagnostics associated with virus isolation and identification techniques that may pose a problem for healthcare authorities in the event of an RVFV epidemic. There is a crucial need for rapid detection and identification of the virus [3], [5].

NanoTrap particles are a novel technology that can address all the critical analytical challenges for pathogen identification and measurement. They are homogenous hydrogel particles of about 800 nanometers in size that have a shell made of polymers of N-isopropylacrylamide (NIPAm) and co-monomers such as acrylic acid (AAc) and allylamine (AA) with cross links of N,N′-methylenebisacrylamide (BIS). This shell can be modified to alter permeability or porosity by increasing or decreasing the percentage of BIS [9], [10]. Charge-based affinity baits are incorporated into the NanoTrap particles by copolymerization and covalent binding to the shell [10]. The NanoTrap particles are temperature- and pH-sensitive, decreasing in size with increased temperature and low pH. The molecular sieving properties of the particles depend on several aspects. The degree of cross-linking within the particles provides inclusion and exclusion of high abundance large molecules (e.g. albumin). Affinity baits further facilitate the capture and concentration of the target protein, and prevent it from exiting the particle. They may be of negative or positive charge, therefore attracting analytes of opposite charge. This was seen in an early experiment performed by Luchini *et al.* where the incubation of particles containing anionic affinity baits captured myoglobin, a protein with a positive charge [9]. Some NanoTrap particles are composed of NIPAm shells, and a few of these shelled NanoTrap particles are also coated with vinyl sulfonic acid (VSA) [11]. NanoTrap particles are able to perform three functions in one step: molecular size sieving, target analyte affinity sequestration, and complete protection of captured analytes from degradation. Furthermore, NanoTrap particles help to bridge the gap between detection and the limits of sensitivity. Mass spectrometry (specifically liquid chromatography coupled with tandem mass spectrometry) is a favored technique for the discovery of candidate biomarkers in biological fluids. However, this technique only accepts a small input volume and complex solutions often lead to decreased sensitivity. The NanoTrap particles concentrate protein analytes in small volumes to effectively amplify the sensitivity of mass spectrometry. In addition, their promiscuity allows for multiple analytes to be harvested from a single sample [9]. Experiments conducted by Luchini *et al.* demonstrated the capture and enrichment of small molecules spiked in complex solutions such as whole blood and serum [9], [10]. A 2011 study by Douglas *et al.* on the detection of Lyme disease demonstrated that NanoTrap particles can improve sensitivity more than 100-fold (over existing methods) as well as lead to 100 percent capture and 100 percent elution yield of low abundance antigens in biofluids. Lyme disease antigens at low abundance were detected in both urine samples as well as from a single infected tick [12].

The current library of commercially available NanoTrap particles has been designed to specifically harvest proteins, peptides, metabolites and small molecules. We hypothesized that NanoTrap particles would be able to capture whole virus through the interaction of the NanoTrap particle with the positively charged residues on the surface of RVFV. Our study demonstrates that NanoTrap particles are capable of capturing whole virus, and can be assayed with both qRT-PCR and plaque assays. Importantly, serial dilution studies and studies in serum indicate that NanoTrap particles increase detection sensitivity at lower viral titers. Furthermore, the virus can be inactivated with either heat or detergent, while the virus captured can still be detected with qRT-PCR. Importantly, the NanoTrap particles protect purified viral RNA as well as stabilize the infectivity of RVFV. The studies described here expand upon the NanoTrap particles repertoire to characterize the capture of viruses.

## Methods

### NanoTrap particles

The NIPAm/AA NanoTrap particles were provided by Ceres Nanoscience, Manassas, VA.

### Cell culture

The Vero cell line (kidney epithelial cells) was grown in Dulbecco's Modified Eagle Medium (DMEM) supplemented with 10% FBS, 1% penicillin/streptomycin, and 1% glutamax (DMEM+++). The J1.1 cell line, which are Jurkat E6.1 suspension cells chronically infected with the LA1 strain of HIV-1, were grown in medium containing advanced RPMI-1640, 10% fetal bovine serum, 1% penicillin/streptomycin, and 1% L-glutamine. All cell lines were cultured in a humidified environment containing 5% CO_2_ at 37°C.

### Viruses

The experiments used a live attenuated vaccine derived from the RVFV ZH548 strain, known as MP-12, which had been isolated in 1977 from a patient with uncomplicated RVFV. The virus was generated by 12 serial passages in MRC5 cells, inducing 25 nucleotide changes across the viral genome [13]. Both RVFV ZH548 and MP12 strains were anonymized. MP12 was propagated by infecting Vero cells at 80–90% confluency at an MOI of 0.1 in DMEM+++. Cell culture medium was collected from the cells when ∼75% cytopathic effect was observed (typically 72 hours post-infection (hpi)). Cell culture medium was centrifuged at 10,000 rpm for 10 minutes to pellet the cellular debris. Cell free-viral supernatants were then filtered using a 0.22 µM filter and viral titer determined by plaque assays. Screening experiments for Venezuelan Equine Encephalitis Virus (VEEV) used the live attenuated vaccine TC-83, which had been derived from the Trinidad donkey (TrD) strain by 83 serial passages in fetal guinea pig hearts. This induced changes at 12 nucleotide positions across the viral genome [14]. The viral supernatant of chronically infected J1.1 cells was used in the HIV-1 screening experiments. The LAV strain of HIV-1 had previously been used to infected Jurkat E6 cells at a multiplicity of infection of 0.1 to 0.01 for 2 hours at 27°C and cultured for two weeks. The cells that survived the cytopathic effects of virus infection were cloned and the supernatant from growth-positive wells were screened for Reverse Transcriptase (RT) activity [15]. The J1.1 cells express viral RNA and proteins at low levels.

### Preparation of commercially available beads

Six commercially available beads - DEAE-Sephadex (Sigma-Aldrich), Dynabeads M-280 Streptavidin (Invitrogen), Sephacryl S-200 beads (GE Healthcare), Biorex 70 Resin (Bio-Rad Laboratories), SP Sephadex C-25 (GE Healthcare), and Bio-gel HTP Hydroxyapatite (Bio-Rad Laboratories) were used to compare their capture to NT53. Each bead was washed four times with water and a 33% percent slurry with water was prepared.

### Standard NanoTrap particle incubation

According to a protocol standardized by Ceres Nanoscience, 100 microliters (µL) of sample was incubated with 75 µL of NanoTrap particles for 30 minutes at room temperature. The sample was centrifuged at 10,000 rpm for 5 minutes and the supernatant was discarded. The pellet was washed with 100 µL of RNase- and DNase-free water four times. The pellet was then resuspended in the appropriate buffer. For lysis of MP12 with NP-40, 1% NP-40 was added to 100 µl of MP12 and incubated at room temperature for 30 minutes. A standard NanoTrap particle incubation was performed followed by a qRT-PCR assay. For the RNase treatment, purified MP12 RNA was treated with RNase A and incubated for one hour at 37°C.

### Plaque assay

Vero cells were plated in 6 well plates at 1.0E+06 cells/ml in order to achieve 100% confluency. After NanoTrap particle incubation and subsequent washes, the pellet was resuspended in 100 µL of supplemented DMEM and serial dilutions performed. Four hundred µL of the serial dilution was added to each well in duplicate and incubated for 1 hour. Three hundred milliliters (mL) of a primary overlay known as the CV mixture containing equal parts 0.6% agarose in distilled water and media containing 2X EMEM, 5% FBS, 1% Minimum Essential Amino Acids, 1% Sodium Pyruvate, and 1% Glutamax was added directly to each well. The cells were fixed with 10% formaldehyde in water after 72 hpi. The cells were stained with 1% Crystal Violet in 20% ethanol and water. After two hours, the crystal violet stain was washed off and the plaques formed were counted to determine the plaque forming units per milliliter (pfu/ml).

### RNA extraction and quantitative real time PCR

After NanoTrap particle incubation and subsequent washes, the pellet was resuspended in 180 µL of lysis/binding solution (Life Technologies) containing guanidinium thiocyanate and incubated on ice for thirty minutes. The samples were spun at 13,000 rpm for 5 minutes at room temperature. The supernatant was transferred to a 96-well plate and RNA extraction was performed with Ambion's MagMax 96-well Viral RNA extraction kit according to manufacturer's instructions. In order to determine the number of viral genomic copies produced, qRT-PCR with viral specific primers was performed using RNA UltraSense One-Step Quantitative RT-PCR System (Life Technologies). The experiment was performed according to a standardized protocol using fifteen µL of master mix containing enzyme mix, 5X reaction mix, 50 mM magnesium sulfate (excluded for VEEV qRT PCR), ROX reference dye, 10 µM TaqMan fluorogenic probe, 10 µM forward primer (AAAGGAACAATGGACTCTGGTCA), and 10 uM reverse primer (CACTTCTTACTACCATGTCCTCCAAT) added to five µL of extracted RNA. The samples were heated at 50°C for 15 minutes, 95°C for 2 minutes, and at 95°C and 60°C for 40 cycles.

### RVFV-spiked animal serum

RVFV was spiked into 100% bovine, sheep, and donkey serum (purchased from Innovative Research) at 1.0E+05 pfu/ml. One mL of spiked serum was used in a standard NanoTrap particle incubation with NT53.

### MP12 inactivation with NP-40 detergent and heat

After a standard NanoTrap incubation was performed with NT53 and RVFV, the samples were incubated at room temperature with 0.1, 0.5, or 1% NP-40 for 1 hour or heated at 57°C for 0.5, 1, or 2 hours. The samples were then analyzed by plaque assays and qRT PCR.

### HIV RT-PCR

A standard NanoTrap particle incubation with one mL of HIV-1 supernatant from infected J1.1 cells and NanoTrap particles was performed. An RNA extraction was performed (as described above). A master mix was then prepared with the following components for a 25 µL reaction: SuperScript III RT/Platinum Taq Mix - 0.5 µL, 2X Reaction Mix with ROX - 12.5 µL, and 0.5 µL of Forward and Reverse Primer Mix (LTR forward primer CGAGCTTGCTACAAGGGACT and LTR reverse primer GAGATTTTCCACACTGACTAAAAG) at 10 µM. Five µL of sample, 6.5 µL of water, and 13.5 µL of master mix were aliquoted out into PCR tubes. The PCR was conducted with the following cycles: 15 min at 50°C (cDNA synthesis), 2 min at 95°C (prime reaction), and 35 cycles at 30 seconds at 95°C (denature), 30 seconds at 51°C (annealing), 30 seconds at 72°C (extend), 72°C for 10 min, and a hold at 4°C. A DNA gel was prepared using 1% agarose powder in 1X TAE buffer with the addition of ethidium bromide for a final concentration of 0.5 µg/µl. The gel was visualized on an ultraviolet transilluminator and the volume of each band was quantified.

## Results

### NanoTrap particles can capture whole virus

**Figure 1:**
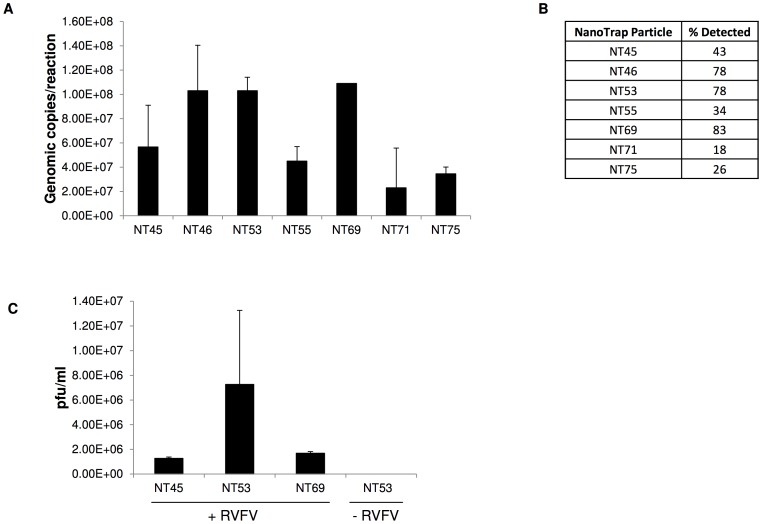
RVFV capture by NanoTrap particles. A) Seven different types of NanoTrap particles were incubated with viral supernatants containing RVFV (1E+7 pfu/ml) for 30 minutes at room temperature and washed 4 times with water. Viral RNA was extracted from the particles with Ambion's MagMax Viral RNA extraction kit and quantitated by qRT-PCR assays. B) Percent detected virus was calculated compared to a sample processed without NanoTrap particle incubation. C) Viral supernatants were incubated with NT46, NT53, and NT69 for 30 minutes at room temperature and washed 4 times with water. Serial dilutions followed by plaque assays were performed to determine if full virus was bound by the particles.

NanoTrap particles have previously been shown to capture proteins. We hypothesized that the RVFV glycoproteins would be capable of interacting with the NanoTrap particles, facilitating capture in a fashion similar to the way in which protein biomarkers interacted with the NanoTrap particles. To test this hypothesis, seven different NanoTrap particles were tested with RVFV. Four NanoTrap particles possessed shells (NT53, NT55, NT69, and NT71) whereas three did not (NT45, NT46, and NT75) (Table 1). We incubated culture supernatants from RVFV-infected Veros with each NanoTrap particle. All seven NanoTrap particles successfully captured virus, averaging 6.8E+07 genomic copies per reaction. Specifically, NT46, NT53, and NT69 captured higher genomic copies than the other NanoTrap particles, with each capturing approximately 1.0E+08 genomic copies per reaction (Figure 1A). This corresponds to 78–83% capture (Figure 1B) of a sample containing a high titer of virus.

**Table 1:** Description of the NanoTrap particles' bait and shell.

NanoTrap ID	Bait	Shell (y/n)	VSA* shell (y/n)
NT45	Reactive red 120+Reactive yellow 86	N	N/A
NT46	Reactive red 120	N	N/A
NT53	Cibacron blue F3GA	Y	N
NT55	Acrylic Acid	Y	N/A
NT69	Cibacron Yellow 3GP	Y	Y
NT71	Cibacron Blue F3GA	Y	Y
NT75	Methyl Acrylate	N	N/A

* Vinyl Sulfonic Acid.

In order to determine if the amplification observed in the qRT-PCR assay was due to the NanoTrap particle capturing intact viral particles or association of viral RNA (presumably due to lysed virus) with the particles, plaque assays were performed. If the particles captured viral RNA or lysed virus, no plaques should be observed. Plaque assays were performed on the three best candidates from the qRT-PCR screening. Captured viruses were not eluted off of the NanoTrap particles, but rather the samples were diluted and added directly to the Vero cells during the plaque assay procedure. We hypothesized that the viral glycoproteins would have a greater affinity for the cellular receptor than the NanoTrap particles and thus would enter the cells. NT53, which contains a cibacron blue bait with a shell, captured infectious RVFV virion six- and four-fold more than NT46 or NT69, respectively (Figure 1C). These plaques were not due to cell death induced by the NanoTrap particles themselves, as NanoTrap particles alone did not produce plaques. Therefore NT53 was chosen for all future experiments with RVFV.

Experiments were performed to determine potential elution methods that would release the virus without affecting the viral particle integrity. It had previously been found that sodium chloride (NaCl) concentrations between 0.5M and 2M could effectively elute various analytes from cibacron blue dyes by disrupting electrostatic interactions between cibacron blue dyes (the bait molecule found within NT53; [16]). We hypothesized that incubating the RVFV-bound NanoTrap particles on ice would allow the particles to swell and, with the aid of vortexing, the virus would disassociate from the NanoTrap particles. Therefore, we tested a NaCl based elution method coupled with an ice-swelling method. Plaque assays were performed to determine the amount of virus eluted from the NanoTrap particles and the amount that remained bound to the particles after a high salt elution. After NT53 incubation with RVFV, the pellets were resuspended in 2.0 M NaCl in DMEM and placed on ice for 30 minutes with vortexing every ten minutes. Both the eluates and pellets were analyzed by plaque assay (Figure S1). The results showed that 5.5% of RVFV was detected after elution with 2.0M NaCl. The addition of NaCl coupled with incubation on ice only slightly released RVFV virions, demonstrating the virus' strong affinity for the NanoTrap particles. However, as seen in Figure 1B, RVFV-bound NanoTrap pellets directly added to Vero cells during the plaque assays procedure were capable of producing plaques. Based on these results, we opted not to elute RVFV from the NanoTrap particles, but rather to add the RVFV bound to the NanoTrap particles directly during the plaque assay procedure.

### Characterization of RVFV NanoTrap particle capture

**Figure 2:**
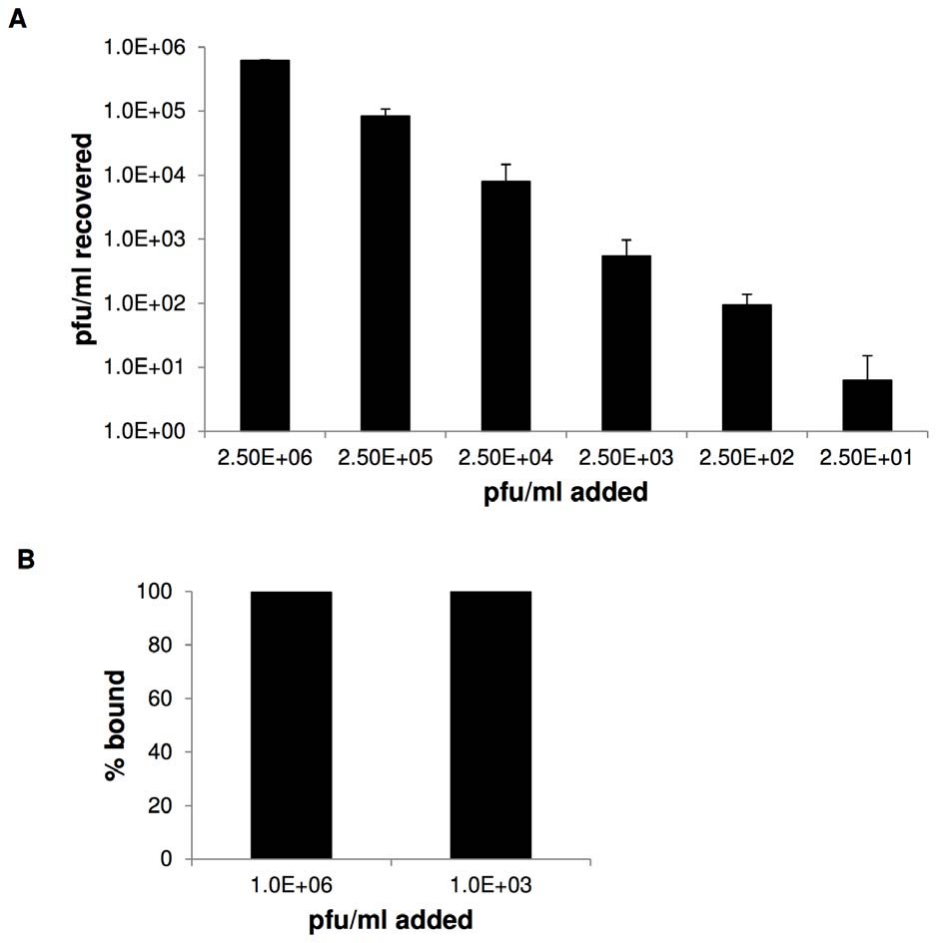
Characterization of RVFV NanoTrap particle capture. A) Viral supernatants were serially diluted (2.5E+6 to 2.5E+1 pfu/ml) and incubated with NT53 for 30 minutes at room temperature. The pellets were washed 4 times with water and then particles were tested in plaque assays to determine if full virus was bound by the particles. B) Viral supernatants at 1.0E+6 and 1.0E+3 pfu/ml were incubated with NT53 for 30 minutes at room temperature. The sample was spun at 10,000 rpm for 5 minutes and the unbound viral supernatant was saved separately. NT53 was washed 4 times with water and then particles were tested in plaque assays to determine how much virus were bound verses unbound by the particles. The percentage of bound virus at 1.0E+6 and 1.0E+3 pfu/ml was graphed.

We next wanted to determine the limit of detection of RVFV in plaque assays with NT53. NT53 was incubated with RVFV at decreasing titers, from 2.5E+6 to 2.5E+1 pfu/ml, and plaque assays performed (Figure 2A). Captured virus was detected down to 2.5E+1 pfu/ml for RVFV. These results show that NanoTrap particles are capable of capturing whole virus even at low viral titers.

We next determined the percentage of RVFV captured by NT53 in comparison to the total input amount. RVFV at 1.0E+6 and 1.0E+3 pfu/ml were added to NT53. At 1.0E+6 pfu/ml, 99.35% of the virus was bound to the NanoTrap particles whereas at 1.0E+03 pfu/ml, ∼100% of the virus appeared bound to the NanoTrap particles (Figure 2B). The results confirm that the NanoTrap particles are efficient at capturing RVFV, especially at a lower titer. Interestingly, the results also suggest that a small volume of RVFV can be captured with NanoTrap particles and then recultured to grow more virus.

### RVFV detection is more sensitive with NanoTrap particle incubation

**Figure 3:**
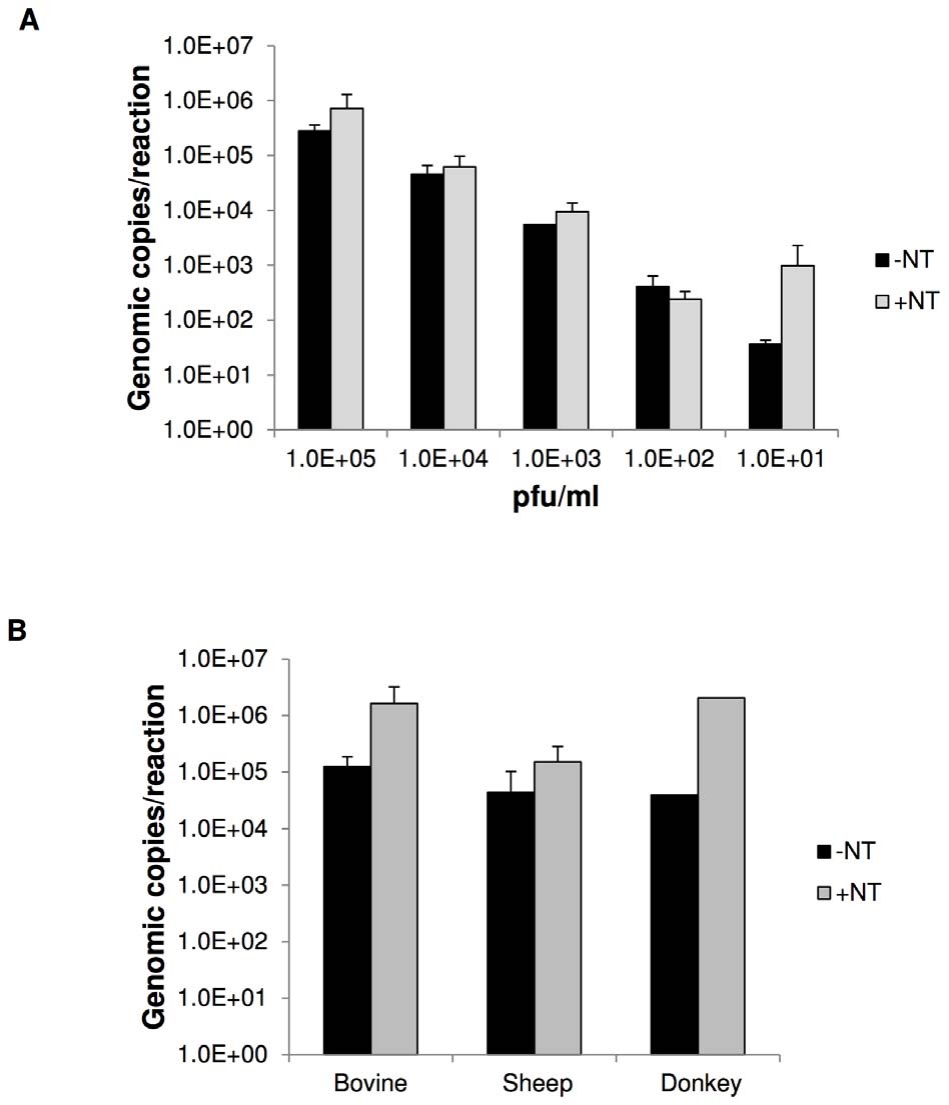
RVFV enrichment with NT53 incubation. A) RVFV was spiked into cell culture media (DMEM+++) at various concentrations from 1.0E+5 to 1.0E+1 pfu/ml. NT53 was added to 1 ml of media and captured according to standardize protocols. The pellet was washed 4 times with water, followed by processing with Ambion's MagMax 96-well Viral RNA extraction kit. RVFV-spiked media without NT53 were processed in parallel. Viral RNA was quantitated by qRT-PCR with viral specific primers. B) RVFV was spiked into 100% bovine, sheep, and donkey serum at 1.0E+5 pfu/ml. NT53 were added to 1 ml of serum and captured according to a standardize protocol. The pellet was washed 4 times, followed by processing with Ambion's MagMax 96-well Viral RNA extraction kit. Serum without NT53 was processed in parallel. Viral RNA was quantitated by qRT-PCR.

In clinical instances of infection, the viral titers in circulation during very early stages after exposure are expected to be low and therefore, hard to detect [17]. We wanted to determine if viral enrichment by the NanoTrap particles (NT53) would enhance detection of RVFV when compared to detection in the absence of enrichment afforded by the NanoTrap. We specifically wanted to see the enrichment potential at lower viral titers when detection would be most difficult. For these assays, we chose to utilize qRT-PCR based detection due to its increased sensitivity over plaque assays. To this end, we spiked RVFV into cell culture media that contained 10% FBS at various concentrations from 1.0E+5 to 1.0E+1 pfu/ml. NT53 was then added to 1 ml of the spiked media. Viral capture with and without NanoTrap particles gave similar yields at higher viral titers (Figure 3A). However, at lower viral titers, there was a significant increase in viral capture with the use of the NanoTrap particles compared to samples without NanoTrap particle capture. There was greater than a 100-fold increase of viral detection with the use of NT53 at 1.0E+1 pfu/ml.

### NanoTrap particle enrichment of RVFV from animal sera

We next wanted to determine if we could capture and enrich virus from a clinically relevant matrix. RVFV was spiked into 100% bovine, donkey, and sheep sera at 1.0E+05 pfu/ml. Incubation of RVFV spiked sera with NT53 resulted in enrichment by 13-, 3-, and 52-fold for bovine, sheep, and donkey sera, respectively (Figure 3B). These results demonstrate that NT53 not only captures but also enriches virus found in complicated matrices such as animal sera. The complex analytes (e.g. albumin) found in the sera are likely excluded by the NanoTrap particles and do not interfere with whole virus capture. However, we speculate that since the serum from each of the three animals contains different analytes, there may be interfering proteins that would lead to the observed enrichment differences.

### NanoTrap particles are capable of capturing inactivated RVFV

**Figure 4:**
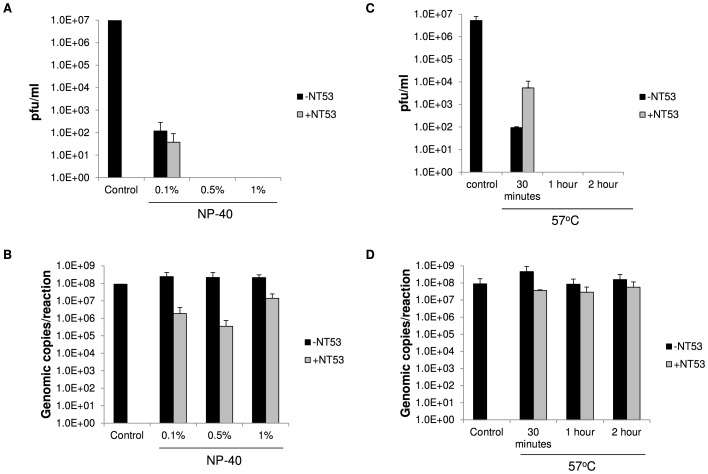
Viral inactivation following NT53 capture. Panels A and B: NT53 was incubated with viral supernatants containing RVFV at 2.7E+8 pfu/ml for 30 minutes at room temperature. Samples were then incubated at room temperature with 0.1, 0.5 or 1% NP-40 for 1 hour (NP-40). Control samples were not treated with NP-40 nor captured with NT53. Samples treated with NP-40 without NT53 were processed in parallel (black bars). Viral inactivation was assayed by plaque assays (A) and viral RNA was extracted from the particles with Ambion's MagMax 96-well Viral RNA extraction kit and quantitated by qRT-PCR (B). Panels C and D: NT53 was incubated with viral supernatants containing RVFV at 2.7E+8 pfu/ml for 30 minutes at room temperature. Samples were then incubated at 57°C for 30 minutes, one hour, or two hours. Control samples were not heat treated nor captured with NT53. Samples heat-treated without NT53 were processed in parallel (black bars). Viral inactivation was assayed by plaque assays (C) and viral RNA was extracted from the particles with Ambion's MagMax 96-well Viral RNA extraction kit and quantitated by qRT-PCR (D).

Viral inactivation is crucial for its transport from the field or a BSL-3 facility to a BSL-2 environment for downstream analysis. However, after inactivation the virus may be susceptible to degradation. Therefore, we wanted to determine if RVFV would remain bound to the NanoTrap particles in an inactivation scenario. After NanoTrap particle incubation with RVFV and subsequent washes, NP-40 detergent was used to inactivate the virus (Figure 4). Plaque assays were performed to confirm viral inactivation. Plaque assays demonstrated that 0.1% NP-40 did not fully inactivate the virus incubated with or without NT53 (Figure 4A). Higher concentrations of NP-40 (0.5% and 1%) fully inactivated RVFV in the presence or absence of NT53. While the plaque assays confirmed inactivity of RVFV, qRT-PCR data demonstrated that RVFV was still captured following NP-40 addition (Figures 4B). In the presence of NP-40 the levels of capture with NT53 were decreased as compared to the controls. This is likely due to the interference of the NanoTrap particle binding to RVFV due to the presence of detergent.

We next tested the ability of NT53 to function in another commonly employed viral inactivation procedure. The samples were heat inactivated at 57°C for three different time points - thirty minutes, one hour, and two hours - and plaque assays were performed to confirm viral inactivation. At thirty minutes approximately 1.0E+4 and 1.0E+2 pfu/ml of RVFV with and without NT53, respectively, were still detectable. Interestingly, RVFV was more resistant to heat inactivation in the presence of NT53, suggesting the NanoTrap particles may have a slight protective effect on the virus. However, complete inactivation was achieved at one hour (Figure 4C). While the plaque assays confirmed inactivation of RVFV, qRT-PCR data demonstrated the ability of NT53 to detect RVFV nucleic acids after heat inactivation (Figure 4D). These data demonstrate that it is possible to fully inactivate the virus before applying downstream assays such as qRT-PCR. Furthermore, these two experiments demonstrate the ability to inactivate a sample and transport it as a non-infectious sample, while still retaining capture.

### NanoTrap particles are capable of capturing and protecting viral RNA

**Figure 5:**
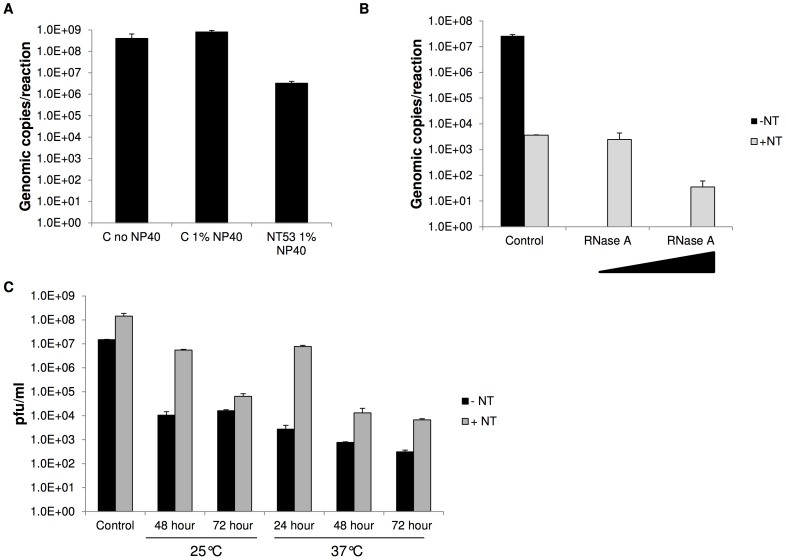
NT53 protects viral RNA from degradation and preserves viral infectivity. A) RVFV was lysed with 1% NP-40 for 30 minutes at room temperature and then incubated with NT53 for 30 minutes at room temperature. The viral RNA was extracted from the particles with Ambion's MagMax 96-well Viral RNA extraction kit and quantitated by qRT-PCR. Samples without NT53 and samples without NP-40 were processed in parallel. B) NT53 was incubated with purified RNA at 2.0E+8 genomic copies for 30 minutes at room temperature. Following water washes, the samples were resuspended in water and treated with 140 or 1400 Units/ml of RNase A. Samples with no RNase A treatment were processed in parallel. The viral RNA was extracted from the particles with Ambion's MagMax 96-well Viral RNA extraction kit and quantitated by qRT-PCR. Samples without NT53 were processed in parallel (black bars). C) RVFV (1.4+E7 pfu/ml) was spiked into 1 ml of bovine serum, NT53 added, and samples incubated at 25°C (for 48 or 72 hr) or 37°C (for 24, 48, or 72 h). Samples without NT53 were processed in parallel. Samples were assayed for viral infectivity by plaque assay. Control samples are samples that were processed immediately for plaque assays with no incubation period.

As we observed viral capture in the presence of NP-40, we hypothesized that the virus was being lysed and the released viral RNA recaptured with the NanoTrap particles. If the NanoTrap particles were not providing protection of the viral RNA, there will be no possibility for any downstream assays using inactivated material, which is a critical step in diagnostics. To test this hypothesis, we first lysed the virus with 1% NP-40 and followed by adding NT53 to the lysed material. Results in Figure 5A indicate that NT53 was able to capture the lysed virus. However, there was a 100-fold decrease in lysed virus with the addition of NT53 compared to the control (no NT53) with and without NP-40. These results mirrored what was observed in Figure 4B, where NT53 was capable of capturing virus to a lesser extent in the presence of NP-40. These results demonstrated that RVFV could be initially inactivated by traditional inactivation methods and then captured with NanoTrap particles. By incubating with the NanoTrap particles, the viral RNA will be protected and hence, can be used for downstream RNA detection.

In order to directly show that NT53 was able to capture and protect viral RNA, we performed a NanoTrap experiment with purified viral RNA. We first incubated NanoTrap particles with purified RNA, and performed qRT-PCR assays. Results in Figure 5B demonstrate that NT53 is capable of capturing purified viral RNA, albeit with less affinity than whole virus capture. NT53 was able to capture 0.01% of the input viral RNA. While we screened the NanoTrap particles for whole virus capture, we did not screen the NanoTrap particles for RVFV viral RNA capture. There is likely a NanoTrap particle that captures viral RNA with greater efficiency than NT53.

As previous studies have shown that proteins captured by NanoTrap particles were protected from trypsin degradation, we next aimed to determine if NanoTrap particle capture could protect viral RNA from RNase degradation [9], [10]. Samples with and without NT53 were treated with RNase A at 140 or 1400 Units/ml and incubated for one hour at 37°C. Interestingly, the RNA incubated with NT53 was protected from RNase A degradation, whereas the RNA controls were subject to complete RNase A degradation (Figure 5B). At 140 Units/ml of RNase A, the captured RNA was detected at the same level as the RNase-untreated sample. Even at a substantially higher RNase concentration (1400 Units/ml), 1% of the viral RNA input was still detected. Our results demonstrate that the NanoTrap particles are capable of capturing and protecting viral RNA from enzymatic degradation.

### NanoTrap particles capture and preserve whole viruses

In some situations, it may be important to retain the infectivity of the captured virus to enable the virus to be propagated for further characterization. Therefore, we evaluated the ability of the NanoTrap particles to capture and preserve the infectivity of RVFV following capture. RVFV was spiked into bovine serum and incubated with or without NT53 at 25°C for 48 or 72 h. In the absence of NT53, the infectivity of RVFV was decreased by ∼3 logs (Figure 5C). In contrast, samples incubated with NT53 displayed only ∼1.5 log decrease by 48 h. Although ∼3.5 log decrease was observed with NT53 at 72 h, this still resulted in increased virus detected as compared to the control samples due to the enrichment afforded by NT53. The infectivity of RVFV was also assessed for samples that were incubated at 37°C, which would likely result in a more rapid decline in viral infectivity and thus 24, 48, and 72 h time points were examined. As suspected, a ∼4 log decrease was observed in samples incubated at 37°C for 24 h without NT53. In contrast, samples captured by NT53 only displayed ∼2 log decrease as compared to the control NT53 sample. At extended time points a further decrease in infectivity was observed with and without NT53, but in all cases a higher amount of infectious virus could be rescued from samples incubated with NT53. Collectively these results demonstrate that NanoTrap particles can capture and preserve viral infectivity up to 72 h at elevated temperatures.

### Comparison of capture efficacy of NanoTrap particles and commercially available beads for RVFV capture

**Figure 6:**
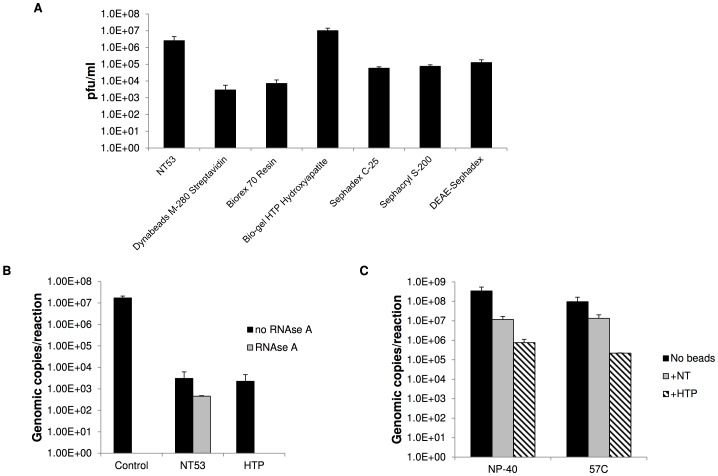
Comparison of capture and RNase degradation protection of commercially available beads with NT53. A) RVFV was incubated with NT53 (1), Dynabeads M-280 Streptavidin (2), Biorex 70 Resin (3), Bio-gel HTP Hydroxyapatite (4), SP Sephadex C-25 (5), Sephacryl S-200 beads (6), or DEAE-Sephadex (7) for 30 minutes at room temperature. The sample was washed 4 times with water and then particles were tested in plaque assays to determine how much virus was bound by the particles. B) NT53 or HTP was incubated with purified RNA at 1.0E+7 genomic copies for 30 minutes at room temperature. Following water washes, the samples were resuspended in water and treated with 380 Units/ml of RNase A. Samples with no RNase A treatment were processed in parallel. The viral RNA was extracted from isolated particles and quantitated by qRT-PCR (black bars). Samples without NT53 were processed in parallel (gray bars). C) NT53 and HTP beads were incubated with viral supernatants containing RVFV at 1.7E+8 pfu/ml for 30 minutes at room temperature. Samples were then inactivated by incubation at 57°C for one hour or incubating in the presence of 1% NP-40 at room temperature for 1 hour. A “no bead” control processed in parallel was included for each condition. Viral RNA was extracted from the particles and quantitated by qRT-PCR.

NanoTrap particles have unique properties not demonstrated in other beads that are used for protein purification and albumin exclusion such as dye baits that make them an ideal candidate in virus capture. Therefore, we wanted to directly compare the ability of other beads to capture RVFV with NT53's RVFV capture capability. The capture of RVFV was tested with NT53 and six commercially available beads used in various assays. DEAE-Sephadex beads are used in ion exchange chromatography for purifying and isolating proteins; Dynabeads M-280 Streptavidin are used for isolating nucleic acids and antibodies; Sephacryl S-200 beads are used to purify protein and macromolecules; Biorex 70 Resin beads are used for purification and fractionation of peptides, proteins, and other cationic molecules; SP Sephadex C-25 beads are used in chromatography to separate and purify protein, polypeptides, and other charged molecules; and Bio-gel HTP Hydroxyapatite beads are used in chromatography to separate and purify proteins, nucleic acids, viruses, and other macromolecules. These calcium phosphate beads work by the cationic interaction of the Ca^2+^ functional groups with the carboxylate residues located on the protein surface and the anionic interaction of the PO_4_^2−^ functional groups with the basic protein residues. RVFV was incubated with each of these beads and plaque assays were performed to determine whole virus capture. Bio-gel HTP Hydroxyapatite (HTP) captured RVFV the most efficiently, averaging 1.0E+7 pfu/ml, while NT53 performed the second best averaging 2.5E+6 pfu/ml (Figure 6A). The other four beads captured RVFV around or below 1.0E+5 pfu/ml.

As we have demonstrated that NT53 not only captures intact RVFV, but can also capture and protect viral RNA from RNase A degradation, we tested the ability of HTP to act in a similar capacity. NT53 was able to fully protect the viral RNA against RNase A degradation and genomic copies for NT53 with and without NT53 treatment were similar. However, HTP beads were unable to provide protection against RNase degradation, and no viral RNA was detected (Figure 6B). A control experiment with RNA alone demonstrated that our RNase treatment was effective. We next compared the ability of NT53 and HTP to capture RVFV during an inactivation scenario. For these experiments NT53 or HTP were added to the samples followed by viral inactivation through treatment with 1% NP-40 or heating at 57°C. The amount of virus captured was quantitated by qRT-PCR (Figure 6C). As was observed in previous experiments, viral inactivation with either NP-40 or heat treatment resulted in some loss of RVFV binding to the NanoTrap (1.2 and 0.8 log, respectively). However, HTP RVFV capture was more dramatically affected, resulting in a 2.5 log decrease with the NP-40 treated samples and a 2.7 log decrease in the heat inactivated samples. Collectively, our experiments demonstrate that while HTP is capable of capturing whole virus, it cannot protect viral RNA against RNase degradation and it displays a reduced ability to capture RVFV during an inactivation scenario. In contrast, NT53 is capable of capturing and protecting RVFV as well as capturing RVFV in samples that have been inactivated by heat or detergent treatment.

### NanoTrap particles are capable of capturing other viruses

**Figure 7:**
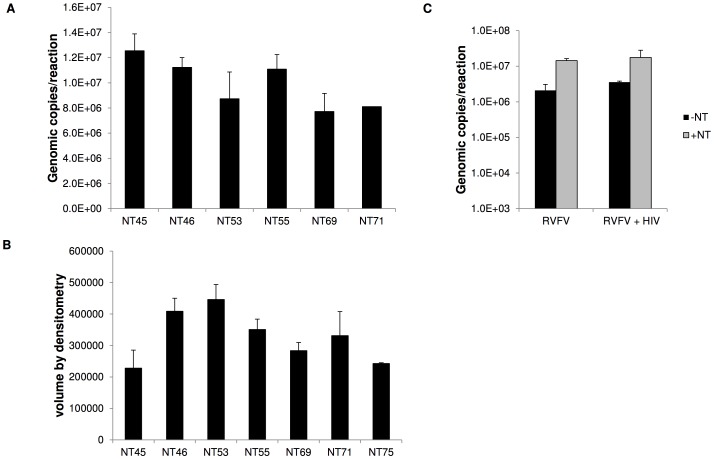
Capture of other viruses with NanoTrap particles. A) Six different types of NanoTrap particles were incubated with viral supernatants containing VEEV for 30 minutes at room temperature and washed 4 times with water. Viral RNA was extracted and quantitated by qRT-PCR assays. B) Seven different types of NanoTrap particles were incubated with 1 ml J1.1 supernatant for 30 minutes at room temperature and washed 4 times with water. The pellets were diluted in 100 ul water and RNA extracted. A cDNA synthesis using 150 ng of each sample was performed and followed by PCR using 10 ul cDNA. A DNA gel was run to determine viral capture. A sample with no reverse transcriptase added and a sample with just water were used as negative controls. The volume of each band was quantified and graphed. C) Bovine serum was spiked with RVFV (1.0E+6 pfu/ml) only or both RVFV (1.0E+6 pfu/ml) and HIV (100 µl of J.1. supernatants). Samples were incubated with NT53 for 30 minutes, viral RNA extracted and quantitated by qRT-PCR. Samples without NT53 were processed in parallel (black bars).

We next asked the question if NanoTrap particles were capable of capturing other viruses. For this, we selected VEEV and HIV-1. VEEV, which at approximately 70 nm in diameter is a smaller virus than RVFV, which is approximately 100 nm in diameter. VEEV viral supernatants were incubated with various NanoTrap particles shown in Table 1 and capture was measured by qRT-PCR. Our data indicated that all six NanoTrap particles successfully captured VEEV, averaging 9.9E+06 genomic copies per reaction (Figure 7A), with a slight preference observed with NT45, NT46, and NT55 capturing 1.3E+07, 1.1E+07, and 1.1E+07 genomic copies per reaction, respectively. NanoTrap particles capture was also tested using HIV-1. HIV-1 supernatants from infected J1.1 cells were incubated with NanoTrap particles. RNA extraction was performed, cDNA was synthesized, and RT-PCR was performed. A semi-quantitative analysis shown in Figure 7B, demonstrated that all seven NanoTrap particles were able to capture HIV-1 with NT46 and NT53 demonstrating the best capture (Figure 7B). These results indicate that NanoTrap particles are capable of capturing multiple viruses.

Viral infections in nature do not occur in isolation and are often accompanied by other co-infections (bacterial and/or viral); therefore we sought to determine if the NanoTrap particles could capture RVFV in a “mixed” infection setting. To this end, bovine serum was spiked with RVFV only or with both RVFV and HIV, followed by NT53 viral capture and quantification as measured by qRT-PCR. Results indicated that NT53 was capable of capturing and enriching RVFV from samples that contained only RVFV or both RVFV and HIV (Figure 7C). Seven-fold enrichment was observed in samples containing RVFV only and 5-fold enrichment from samples containing both RVFV and HIV. These data provide evidence that the NanoTrap particles could be used with clinical samples.

Therefore, in conclusion, the results demonstrate that NanoTrap particles can capture and enrich RVFV from both cell culture media and clinically relevant matrices. The captured virus can then be inactivated and viral RNA protected from enzymatic degradation. The bound RVFV can be eluted off the NanoTrap particles, and used in downstream assays such as plaque assays and qRT-PCR. Furthermore, NanoTrap capture can be extended to other viruses as well, including VEEV and HIV.

## Discussion

Rift Valley Fever Virus is a zoonotic virus that primarily affects livestock but has the potential to cause severe disease in humans. RVFV has led to outbreaks in Egypt and the Arabian Peninsula with the potential to spread to the United States and Europe. Changes in climate, travel, and trade have made RVFV an emerging disease that can have deadly economic and social consequences. Furthermore, RVFV is of biodefense interest due to its potential spread via aerosolization. There are currently no FDA-approved vaccines, so there is a reliance on sensitive and specific diagnostics early on in infection.

The current state of RVFV diagnostics includes virus isolation, nucleic acid techniques, and antibody detection. Current RT-PCR-based assays require a critical amount of the virus circulating in the system. This can lead to misdiagnosis, especially false-negative results, of the disease early on in infection. In contrast, our results demonstrate the ability of NanoTrap particles to enrich for RVFV from both cell culture supernatants as well as more complex matrices such as animal serum. The capability of NanoTrap particles to enrich virus is crucial early on in infection during which the virus can go undetected using other diagnostic methods. In our serum sample studies we noted different levels of enrichment depending on the source of the serum. For example, NanoTrap particles incubated in donkey serum resulted in a 52-fold increase in RVFV detection sensitivity, whereas incubation in sheep serum only displayed a 3-fold increase. The presence of other analytes found in serum may be competing for capture with NanoTrap particles, which likely will differ between species as well as between individual animals. Importantly we have also demonstrated the ability to capture RVFV in samples that also contained HIV. This is an important area of investigation, as clinical samples will likely contain multiple pathogens, providing further competition for NanoTrap binding. We hope to extend our spiked serum sample studies to experiments with serum samples taken from animals exposed to RVFV and human clinical samples. These studies will allow further optimization of the NanoTrap particle collection. In our current study we used very stringent wash conditions to ensure that the virus captured was tightly bound to the NanoTrap particles. In clinical samples, it may be necessary to decrease the number of wash steps to ensure that the maximum amount of virus is being captured from more complex samples. Alternatively, different wash buffers (altering salt and detergent concentrations) could be utilized to allow more selective binding of analytes. Due to the complex nature of clinical samples, it may also be necessary to increase the amount of NanoTrap particles added to prevent saturation. Nonetheless, our studies provide an important first step in the application of NanoTrap particles as a sample preparation and enrichment process to improve diagnostics from serum samples.

One important advantage of utilizing NanoTrap particles is their ability to protect analytes from degradation. Previous studies have indicated that protein captured by NanoTrap particles are protected from trypsin degradation [9], [10]. In these experiments PDGF was incubated with an excess of trypsin. Following incubation the majority of the trypsin was found outside of the NanoTrap particles. However, even though some of the trypsin entered the particles, PDGF was completely protected from degradation. In the current study we extend these findings to demonstrate that viral RNA was protected from degradation in the presence of RNase A. Sample preservation is critical for stabilization of sample integrity both during field collection and during transported to diagnostic facilities. Bio-gel HTP Hydroxyapitite, while able to capture RVFV was unable to protect viral RNA from degradation, further demonstrating the advantage of using NanoTrap particles over other commercially available chromatography beads.

Another critical aspect of the NanoTrap particles is the ability to collect viral samples and inactivate them to render them non-infectious, while still retaining the ability to detect the analyte of interest. This was demonstrated by captured of RVFV by NT53 followed by inactivation of RVFV with NP-40 (determined by plaque assays). Following inactivation, viral genetic material was still detected with qRT PCR and to a higher level than that observed with HTP beads. This is of particular importance in the transport of RVFV from a BSL-3 environment or field sample collection setting to a BSL-2 laboratory for diagnostic testing. Given that BSL-3 laboratories are both difficult to access and work in a BSL-3 environment is time-consuming and expensive, the inactivation method will allow for fewer lapses in time between obtaining the samples and the determining the results. In addition, as the NanoTrap particles allow the capture of the whole virus, the samples can then be analyzed with a variety of downstream analysis methods such as ELISA for the nucleoprotein of RVFV, western blotting, plaque assays, and qRT-PCR.

The exact mechanism of NanoTrap binding to RVFV is unclear at this point. We hypothesize the NanoTrap particle capture is occurring through interactions with RVFV's glycoproteins, Gn and Gc. Gn and Gc are the only viral proteins available for capture by virtue of being exposed on the outside of the virion. The fact that the binding observed with HTP beads slightly exceeded NT53 in binding RVFV may provide insight into the mechanism of binding. HTP is known to bind primarily through electrostatic interactions and similarly, Cibacron blue, the affinity bait component of NT53 binds through electrostatic, hydrophobic or a combination of surface and electrostatic interactions. Based on these results, we expect that electrostatic interactions may provide the dominant mode of NT53 binding to RVFV. Due to the size of the virus (90–100 nm) as compared to the size of the NanoTrap particles (800 nm), it is unclear if the viruses are entering inside the core of the NanoTrap particle or binding to the outside of the NanoTrap particles. We have observed preferentially binding of RVFV with NanoTrap particles containing Cibracon blue baits, suggesting that the bait plays at least a partial role in the binding. Even if RVFV binding is partially or primarily found on the surface of the NanoTrap particles, the particles provide a unique advantage over other commercially available beads, which is sequestration of analytes within the NanoTrap particles. This is important as many enzymes (proteases, RNase,etc.) found in serum can rapidly digest protein and RNA. However, the NanoTrap particles can bind to small molecular weight proteins (such as trypsin) rendering them inactive [9], thereby providing protection for other proteins and/or viruses captured by the NanoTrap particles.

NanoTrap particles can be engineered with increased pore sizes to facilitate capture of RVFV inside the NanoTrap particles. This approach has the added advantage of ensuring capture within the particles themselves, which would be predicted to further increase the stability of the virus as well as increase the viral binding capacity of the NanoTrap particles. One potential disadvantage of larger pores sizes would be the loss of some of the sieve sieving capabilities of the particles. The size sieving is important for more complex samples (whole blood, sera, etc.), which would benefit from the ability of the NanoTrap particles to enrich for certain analytes (i.e. viruses) while excluding high abundant proteins such as BSA, which may interfere with downstream assays or mask lower abundant molecules such as low levels of viruses. However, it may be possible to obtain a balance of larger pores with efficient size sieving if the appropriate level of cross-linking could be achieved. This is an active area of research and warrants further investigation.

Our results demonstrated that NanoTrap particle capture was not limited to RVFV, but could be extended to other viruses including VEEV and HIV. All three of these viruses are enveloped RNA viruses. Future studies will focus on the capture and enrichment of different viral classes, including DNA viruses and non-enveloped viruses. That capture of a wide range of viruses is especially important when multiple viruses cause the same type of disease and/or the symptoms of infection are very general. For example, respiratory infections can be attributed to multiple pathogens, including Influenza A and B viruses, Coronaviruses, and Adenoviruses. For this type of application, the promiscuity of the NanoTrap particles is particularly important, as it will allow the capture and enrichment of multiple viruses from the same sample. Therefore, we believe that increasing sensitivity for respiratory viral infections is an important diagnostic issue that the NanoTrap particles could address.

In conclusion, our results have demonstrated that NanoTrap particles are able to capture and enrich whole virus. While other commercially available beads can also capture virus, only NanoTrap particles are capable of protecting the integrity of the virus after inactivation with detergent or exposure to RNase A. However, further research is needed to determine the exact mechanism by which the NanoTrap particles capture and protect the virus.

## Supporting Information

Figure S1 **Elution with NaCl as demonstrated by plaque assays.** RVFV supernatants were incubated with NT53 for 30 minutes at room temperature. The sample was spun at 10,000 rpm for 5 minutes. NT53 was washed 4 times with water and then particles were eluted with DMEM+2M NaCl and incubated on ice for 30 minutes with vortexing every 10 minutes. The particles were tested in plaque assays to determine how much virus was eluted off of the particles.
(TIF)
